# Construction of an 11-microRNA-based signature and a prognostic nomogram to predict the overall survival of head and neck squamous cell carcinoma patients

**DOI:** 10.1186/s12864-020-07104-w

**Published:** 2020-10-06

**Authors:** Yusheng Huang, Zhiguo Liu, Limei Zhong, Yi Wen, Qixiang Ye, Donglin Cao, Peiwu Li, Yufeng Liu

**Affiliations:** 1 The First Affiliated Hospital, Guangzhou University of Chinese Medicine, No. 12 Airport Road, Baiyun District, Guangzhou, 510407 China; 2 Department of Oral and Maxillofacial Surgery, Guangdong Provincial Key Laboratory of Stomatology, Guanghua School of Stomatology, Sun Yat-Sen University, 56 Lingyuanxi Road, Guangzhou, China; 3 Department of Laboratory Medicine, Guangdong Second Provincial General Hospital, Guangzhou, China; 4 Department of Pediatrics, Shenzhen Hospital of Integrated Traditional Chinese and Western Medicine, Shenzhen, China; 5 Guangzhou First People’s Hospital, Guangzhou, 510000 China

**Keywords:** microRNA, Head and neck squamous cell carcinoma, Overall survival, Risk signature, Nomogram

## Abstract

**Background:**

Head and neck squamous cell carcinoma (HNSCC) is a fatal malignancy owing to the lack of effective tools to predict overall survival (OS). MicroRNAs (miRNAs) play an important role in HNSCC occurrence, development, invasion and metastasis, significantly affecting the OS of patients. Thus, the construction of miRNA-based risk signatures and nomograms is desirable to predict the OS of patients with HNSCC. Accordingly, in the present study, miRNA sequencing data of 71 HNSCC and 13 normal samples downloaded from The Cancer Genome Atlas (TCGA) were screened to identify differentially expressed miRNAs (DEMs) between HNSCC patients and normal controls. Based on the exclusion criteria, the clinical information and miRNA sequencing data of 67 HNSCC samples were selected and used to establish a miRNA-based signature and a prognostic nomogram. Forty-three HNSCC samples were assigned to an internal validation cohort for verifying the credibility and accuracy of the primary cohort. Gene Ontology (GO) and Kyoto Encyclopedia of Genes and Genomes (KEGG) analyses were performed to explore the functions of 11 miRNA target genes.

**Results:**

In total, 11 DEMs were successfully identified. An 11-miRNA risk signature and a prognostic nomogram were constructed based on the expression levels of these 11 DEMs and clinical information. The signature and nomogram were further validated by calculating the C-index, area under the curve (AUC) in receiver-operating characteristic curve analysis, and calibration curves, which revealed their promising performance. The results of the internal validation cohort shown the reliable predictive accuracy both of the miRNA-based signature and the prognostic nomogram. GO and KEGG analyses revealed that a mass of signal pathways participated in HNSCC proliferation and metastasis.

**Conclusion:**

Overall, we constructed an 11-miRNA-based signature and a prognostic nomogram with excellent accuracy for predicting the OS of patients with HNSCC.

## Background

Head and neck squamous cell carcinoma (HNSCC), the sixth most common and eighth most fatal malignancy worldwide [1] is an epithelial tumor arising from the oral cavity, nasal cavity, larynx, hypopharynx, and pharynx. Excessive consumption of tobacco and alcohol is considered a major risk factor for the occurrence and development of HNSCC [2]. In addition, human papillomavirus infection was recently confirmed as an important factor underlying HNSCC progression [2, 3]. Despite the rapid development in examination techniques, HNSCC is generally detected at advanced stages owing to the lack of awareness of regular inspections and no or mild symptoms at early stages. Hence, HNSCC is associated with high mortality [4]. Many patients with HNSCC develop distant metastases within 5 years of receiving comprehensive and systematic chemotherapy [2]. This serves as a significant contributor to death. Thus, improvement in the screening rate of early tumors may be useful as an effective measure to reduce HNSCC-related mortality.

MicroRNAs (miRNAs) are short nonprotein-coding RNAs involved in post-transcriptional regulation of protein-coding gene expression via binding to the 3′-untranslated regions of target mRNAs [5]. miRNAs participate in various physiological and pathological activities in the human body, including cell development, differentiation, cycle regulation, and apoptosis [6, 7]. Several studies have reported the potential diagnostic or prognostic roles of miRNAs in HNSCC, including those of *miR-let-7a-5p*, *miR-3928*, *miR-936*, *miR-383*, *miR-615*, *miR-877*, *miR-9-5p*, and *miR-9-3p* [8–11]. The suppression of *miR-30a* and *miR-379* expression could facilitate the oncogenic activity via upregulating the *DNMT3B* expression and activating the hypermethylation of *ADHFE1* and *ALDH1A* genes in oral squamous cell carcinoma [12]. In addition, *circ-0000495* has been shown to sponge *miR-488-3p* expression and epigenetically silence *TROP2* expression, resulting in the weakening of the proliferative capacity of HNSCC [13]. Thus, the functions of miRNAs affect HNSCC generation, development, and metastasis and are highly associated with the overall survival (OS) of patients with HNSCC.

In the present study, we investigated the miRNAs that were closely bound up with the OS of patients with HNSCC. A miRNA-based signature based on differentially expressed miRNAs (DEMs) as well as a novel miRNA-based prognostic model were constructed to reliably predict the OS of HNSCC patients and provide an important tool for clinicians to improve treatment regimens.

## Results

### Identification of DEMs associated with HNSCC patients

**Fig. 1:**
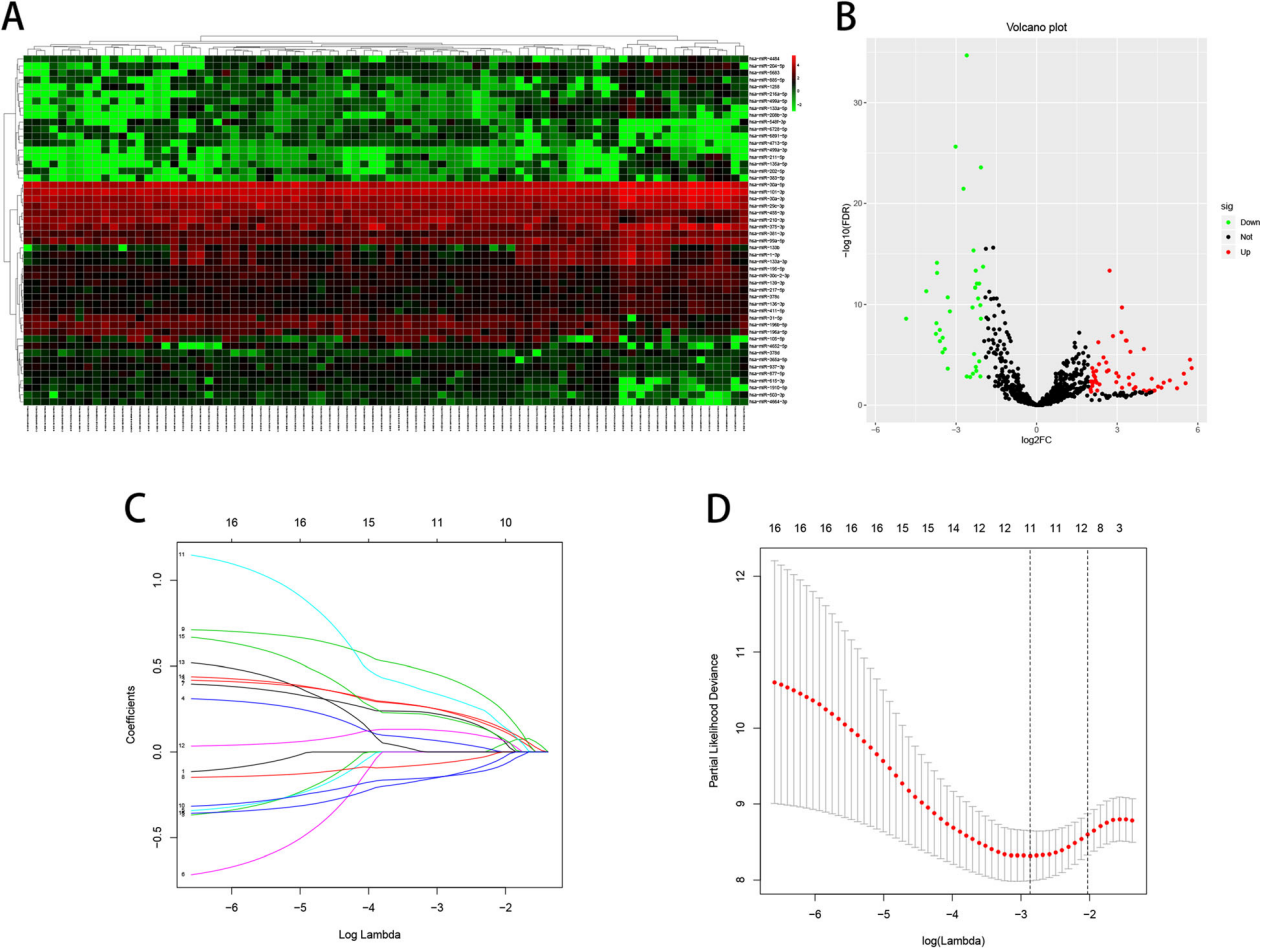
Identification of DEMs associated with HNSCC patients. **a**, the heatmap of 50 DEMs. **b**, the volcano plot of 797 miRNAs. **c** and **d**, the LASSO Cox regression analysis of 16 miRNAs, and coefficients of 11miRNAs ≠ 0 in the **c** when dotted line in the **d** cross to the **c**

Raw HNSCC datasets, consisting of 71 HNSCC samples and 13 normal samples, were downloaded from The Cancer Genome Atlas (TCGA) database. In total, 797 miRNAs were acquired after eliminating those with expression levels < 1. In the heatmap **(**Fig. 1), the expression levels of 50 miRNAs were visually displayed. The differential expression of 797 miRNAs was visually observed using a volcano plot **(**Fig. 1). Of these, 90 miRNAs with |log2FC| ≥ 2 and an adjusted *P*-value < 0.05, including 54 upregulated and 36 downregulated miRNAs, showed significant differential expression. After eliminating the miRNAs detected in 13 normal samples and 4 patients, 90 DEMs were subjected to a univariate Cox proportional hazard regression (CPHR) analysis to determine the independent prognostic impact of individual genes. The results of the univariate CPHR analysis showed that 16 DEMs had the capacity to influence prognosis. Next, these 16 DEMs were subjected to LASSO Cox analysis, and a LASSO Cox regression model with a 10-fold cross validation result was proposed **(**Fig. 1c and d**)**. In total, 11 DEMs were identified the close correlation with the prognosis of patients with HNSCC (Table 1).

**Table 1:** LASSO regression analysis of miRNAs

miRNA	Coefficient	Type	Down−/upregulated
***hsa-miR-204-5p***	0.236292	Risky	Up
***hsa-miR-499a-5p***	0.059085	Risky	Up
***hsa-miR-498-5p***	0.211516	Risky	Up
***hsa-miR-155-3p***	−0.061566	Protective	Down
***hsa-miR-4714-3p***	0.434481	Risky	Up
***hsa-miR-365a-5p***	−0.141218	Protective	Down
***hsa-miR-30a-5p***	0.321480	Risky	Up
***hsa-miR-1-5p***	0.122628	Risky	Up
***hsa-miR-548f-3p***	0.240339	Risky	Up
***hsa-miR-518a-3p***	0.196145	Risky	Up
***hsa-miR-196b-5p***	−0.140244	Protective	Down

### Construction of a risk signature

**Fig. 2:**
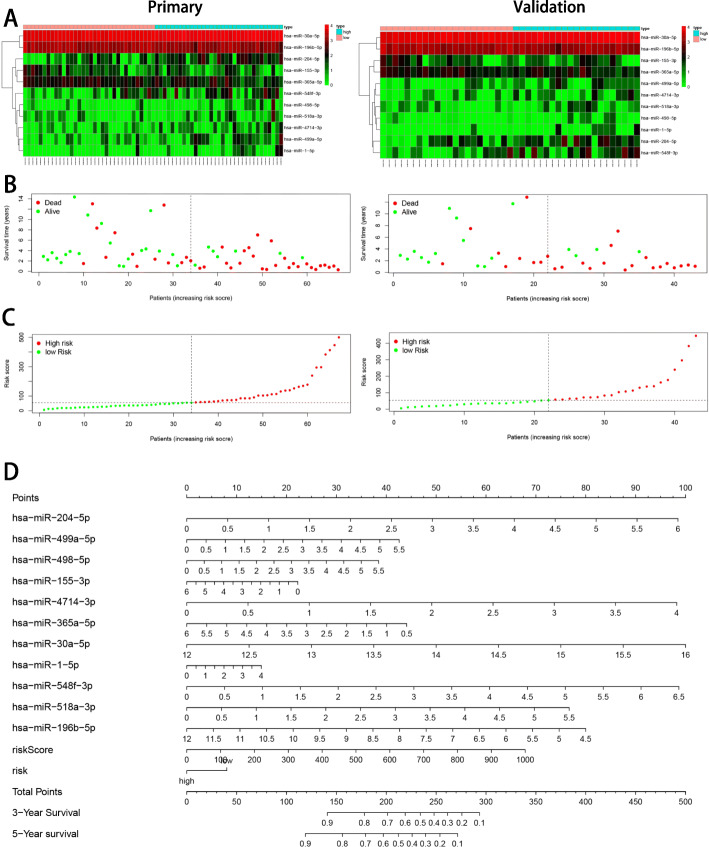
11 miRNAs-based risk signature construction. **a**, the heatmap of 11 miRNAs. **b**, the distribution of OS. **c**, the distribution of risk score. **d**, the prognostic nomogram based on risk signature and 11 miRNAs was used to predict 3- and 5-year OS of patients with HNSCC

The 11 DEMs verified from the LASSO regression analysis were used to generate a risk signature as per the following formula: Risk score = (0.236 × expression _*miR-204-5p*_) + (0.059 × expression _*miR-499a-5p*_) + (0.212 × expression _*miR-498-5p*_) − (0.062 × expression _*miR-155-3p*_) + (0.434 × expression _*miR-4714-3p*_) − (0.141 × expression _*miR-365a-5p*_) + (0.321 × expression _*miR-30a-5p*_) + (0.123 × expression _*miR-1-5p*_) + (0.240 × expression _*miR-548f-3p*_) + (0.196 × expression _*miR-518a-3p*_) − (0.140 × expression _*miR-196b-5p*_). Patients with HNSCC were distributed into high-risk and low-risk groups according to the median of risk score value. The new heatmap generated (Fig. 2a) clearly revealed the differences in the expression levels of the 13 DEMs between high-risk and low-risk groups. Eight DEMs (*miR-204-5p*, *miR-499a-5p*, *miR-498-5p*, *miR-4714-3p*, *miR-30a-5p*, *miR-1-5p*, *miR-548f-3p*, and *miR-518a-3p*) in the primary and internal validation cohorts showed higher expression in the high-risk group than that in the low-risk group. Contrarily, *miR-155-3p*, *miR-365a-5p*, and *miR-196b-5p* were overexpressed in the low-risk group, suggesting that they might might function as tumor suppressors. The survival status and risk score distribution analyses further demonstrated the high risk in the high-risk group **(**Fig. 2b and c). We established a prognostic nomogram associated with 11 DEMs (Fig. 2d) and found that *miR-4714-3p*, *miR-30a-5p*, and *miR-548f-3p* strongly affected the OS of patients.

### Estimation of the reliability of the risk signature

**Fig. 3:**
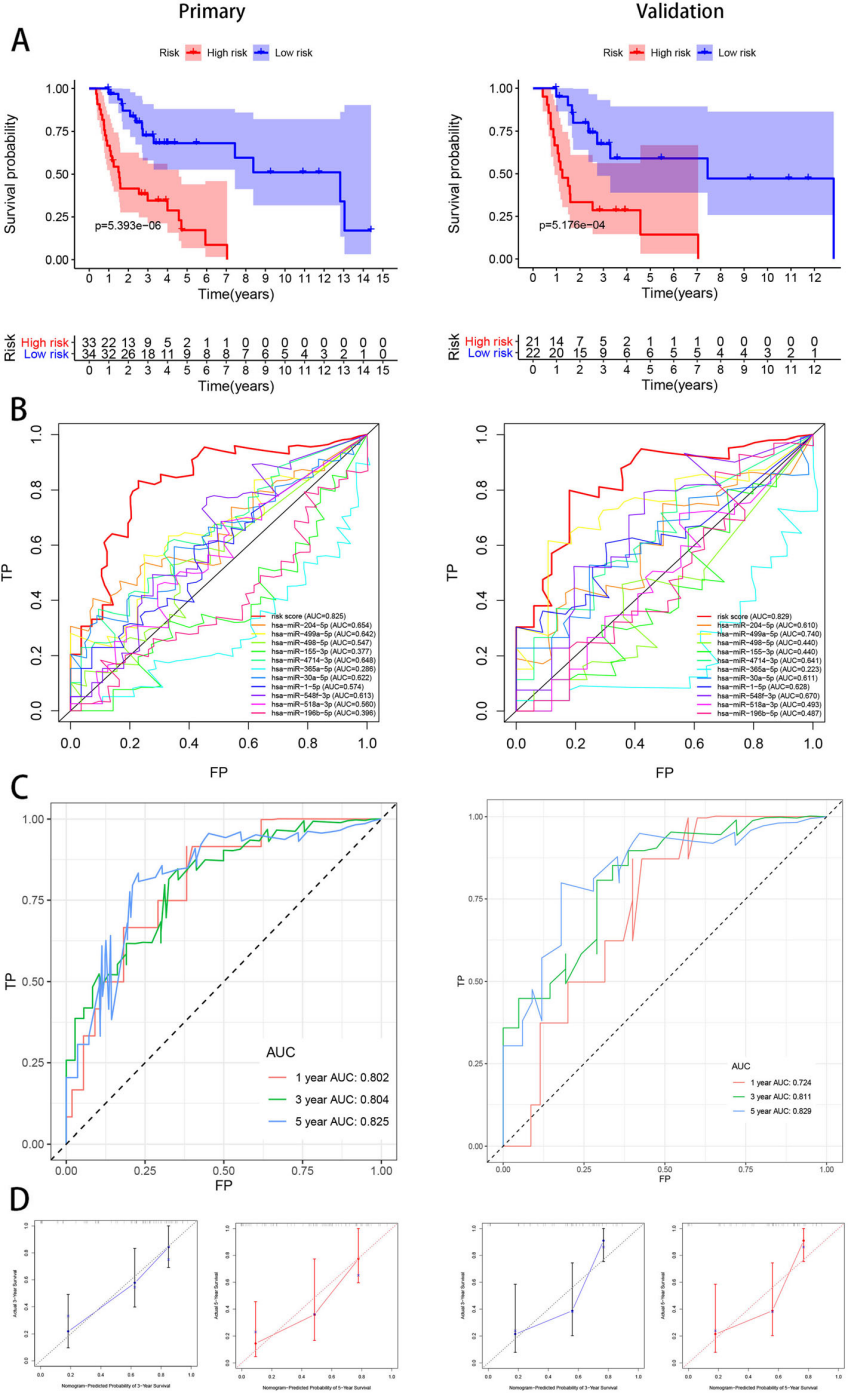
11 miRNAs-based risk signature evaluation. **a**, the Kaplan-meier survival analysis revealed the difference of survival rate between high and low risk group. **b**, AUC in ROC analysis for 11 DEMs and risk signature at 5-years survival time. **c**, 1-, 3- and 5-year AUC in ROC analysis. **d**, Calibration curves of risk signature used for evaluating the 3- and 5- year AUC

To estimate the reliability of the risk signature established herein, a Kaplan–Meier survival analysis (Fig. 3a) was performed. The result of this analysis revealed the shorter OS for patients from the high-risk group than for those from the low-risk group both in the primary (*P* = 5.393e− 06) and internal validation cohorts (*P* = 5.176e− 04). In addition, the area under the curve (AUC) value of the risk signature for 5-year OS had reliable predictive accuracy (Fig. 3b). In the primary cohort, the AUC values of the receiver-operating characteristic (ROC) curve analysis for the risk signature for 1-, 3-, and 5-year OS were 0.802, 0.804, and 0.825, respectively. These values were reported to be 0.724, 0.811, and 0.829 for 1-, 3-, and 5-year OS in the internal validation cohort (Fig. 3c). The calibration curves of the risk signature in the two cohorts revealed excellent agreement between the expected and actual outcomes for 3- and 5-year OS (Fig. 3d). Furthermore, the C-index value for both the primary and internal cohorts was 0.77, indicating considerable accuracy.

### Establishment and evaluation of a nomogram

**Fig. 4:**
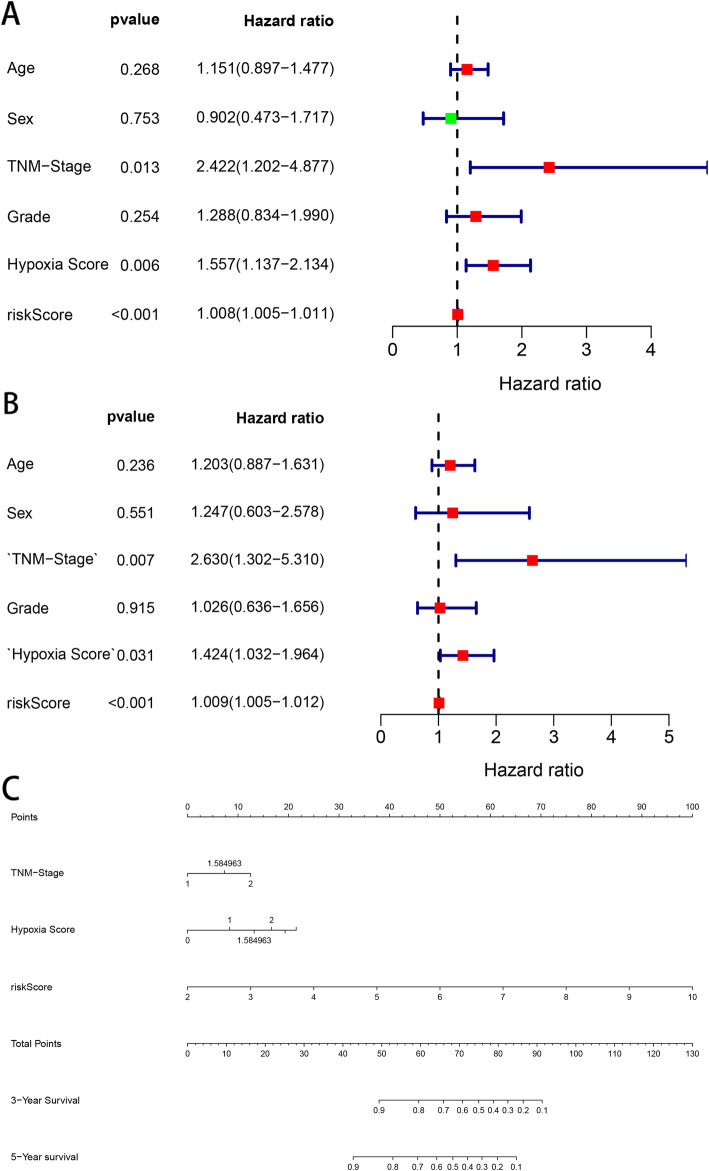
Univariate and multivariate analysis were used to verified factors related HNSCC patient. **a** and **b**, the univariate CPHR analysis and the multivariate CPHR analysis were using to estimate whether these clinical factors and risk signature are independent prognostic variables or not. **c**, the prognostic nomogram established by risk signature, TMN-stage and hypoxia score was used to predict 3- and 5-year OS of patients with HNSCC

**Fig. 5:**
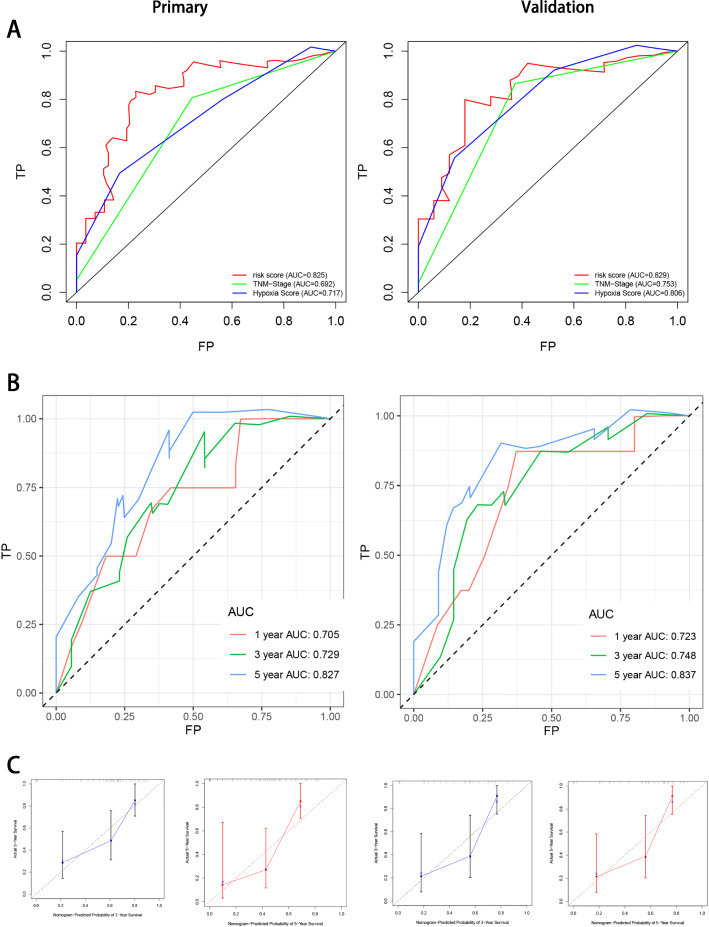
The novel nomogram validation. **a**, AUC of risk signature, TMN-stage and hypoxia score in ROC analysis were calculated at 5-year survival time. **b**, AUC of risk signature in ROC analysis were calculated at 1-, 3- and 5-years survival time. **c**, Calibration curves of risk signature used for evaluating the 3- and 5- year AUC

The clinical information, including age, sex, TNM stage and grade, and hypoxia score, was remarkably associated with the OS of patients with HNSCC (Table 2). Univariate and multivariate CPHR analyses were carried out to obtain information and risk scores for the primary cohort (Fig. 4a and b). In the primary cohort, we proved those factors were independent prognostic variables of OS, including TNM stage, hypoxia score, and risk score. Further, a prognostic nomogram was established using three independent prognostic variables (Fig. 4c). The miRNA signature was more effective in predicting the OS of HNSCC patients, followed by TNM stage and hypoxia score. Further, the AUC values of the ROC curves of the two independent prognostic variables demonstrated that each variable had credible predictive accuracy, especially the miRNA signature (Fig. 5a). The AUC values of the ROC analysis for nomogram were 0.705, 0.729, and 0.827 at 1-, 3-, and 5-year OS, respectively, for the primary cohort, and 0.723, 0.748, and 0.837 at 1-, 3-, and 5-year OS, respectively, for the internal validation cohort (Fig. 5b). To assess the calibration capability of this prognostic model, we established calibration curves and found that the predicted and actual survival in the two cohorts corresponded using this prognostic model (Fig. 5c). The C-index values of the nomogram were 0.776 and 0.744 in the primary and internal validation cohorts, respectively.

**Table 2:** Clinicopathologic characteristics of HNSCC patients in two cohorts

Variables	Primary cohort		Validation cohort	
	***N*** = 67	%	***N*** = 43	%
**Age**				
≤ 60	23	34.33	14	32.56
> 60	44	65.67	29	67.44
**Sex**				
Female	25	37.31	15	34.88
Male	42	62.68	28	65.12
**TNM stage**				
I	2	2.99	2	4.65
II	18	26.87	10	23.26
III	10	14.93	7	16.28
IV	32	47.76	21	48.84
NA	5	7.46	3	6.98
**neoplasm histologic grade**				
1	12	17.91	9	20.93
2	39	58.21	23	53.49
3	14	20.90	10	23.26
NA	2	2.99	1	2.33
**Ragnum Hypoxia Score**				
<0	1	1.49	1	2.33
0–9	10	14.93	5	11.63
10–19	49	73.13	31	72.09
≥ 20	7	10.45	6	13.95
**Survival status**				
Alive	28	41.79	17	39.53
Dead	39	58.21	26	60.47

### Target genes functional enrichment analysis

**Fig. 6:**
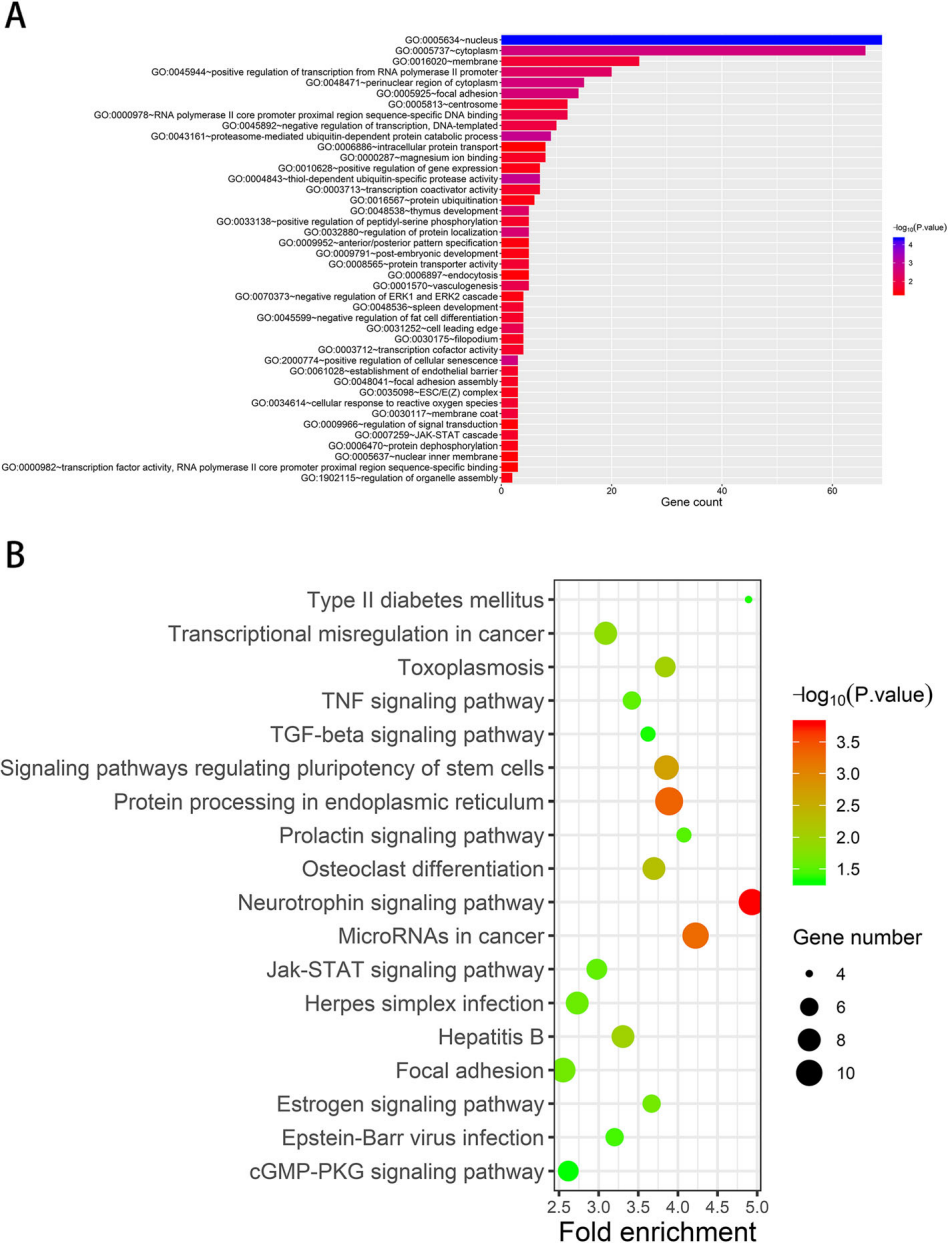
Gene set enrichment analysis. **a**, GO enrichment analyses revealed 11 miRNAs potentially regulated HNSCC progression via 42 categories. **b**, KEGG enrichment analyses revealed 18 signal pathways taken part in HNSCC progression

We predicted the corresponding target genes using three independent databases to confirm the potential biological functions of the 11 DEMs. In total, 38,191 target genes were detected, of which 305 genes were overlapping. Thus, these overlapping genes potentially modulated by the 11 DEMs were subjected to GO and KEGG enrichment analyses. Based on the criterion of a *P*-value < 0.05, 42 categories, including nucleus, cytoplasm, and membrane, showed a strong influence on the occurrence and development of cancer (Fig. 6a). In addition, the KEGG enrichment analysis revealed the multiple pathways that play key roles in HNSCC progression, especially the neurotrophin signaling pathway and protein processing in endoplasmic reticulum (Fig. 6b).

## Discussion

Many reports have suggested the participation of miRNAs in different pathological processes related to HNSCC [8, 13]. However, there is no single representative miRNA that predicts the OS of patients with HNSCC. In the present study, we collected information on patients with HNSCC and evaluated their miRNA expression levels to systematically analyze the miRNAs and clinical characteristics associated with their OS.

We performed the CPHR and LASSO regression analyses and identified 11 DEMs, including *miR-204-5p*, *miR-499a-5p*, *miR-498-5p*, *miR-155-3p*, *miR-4714-3p*, *miR-365a-5p*, *miR-30a-5p*, *miR-1-5p*, *miR-548f-3p*, *miR-518a-3p*, and *miR-196b-5p*, that were confirmed to influence the OS of patients with HNSCC. Kimura et al. conducted a microarray analysis and found that the expression of *miR-30a-5p* was upregulated both in HNSCC and esophagus squamous cell carcinoma cell lines compared to that in normal squamous epithelial cell lines [14]. Another study reported the significantly upregulated expression of *miR-30a-5p* and *miR-769-5p* in the plasma of patients with oral squamous cell carcinoma (OSCC) and their effectiveness as minimally invasive biomarkers for OSCC diagnosis [15]. However, the function of *miR-30a-5p* in patients with HNSCC is yet unclear. A previous study found that *miR-769-5p* promotes the growth of glioma cells through the suppression of *KMT2A* [16] and could be useful as a therapeutic target for glioma treatment. The expression of *miR-518a-3p* has been found to be upregulated in hepatocellular carcinoma, as demonstrated by Taqman low-density miRNA array and quantitative real-time polymerase chain reaction [17, 18], indicative of its tumor-inducing properties. In addition, *miR-518a-3p* has been shown to promote the proliferation, migration, and invasion of choriocarcinoma cells; knockdown of *miR-518a-3p* expression has been found to induce S phase arrest in choriocarcinoma cells [19]. *miR-155-3p* expression downregulation in breast cancer is related to resistance to tumor invasion and metastasis and reduction in paclitaxel resistance [20]. *miR-365a-5p* inhibits the viability, colony formation, migration, and invasion of non-small cell lung cancer cells by negatively regulating Pellino E3 ubiquitin protein ligase family member 3 (*PELI3*) [21], and *PELI3* silencing promotes the *miR-365a-5p*-mediated suppression of pro-tumoral effects. However, the expression of *miR-499a-5p* was down-regulated in OSCC tissues [22]. During glioma progression, *miR-499a-5p* exerts tumor-suppressive effects by regulating the formation of vasculogenic mimicry [23]. In addition, the overexpression of *miR-499a-5p* can markedly inhibit tumor cell proliferation, invasion, and migration [24]. The expression of *miR-204-5p* is suppressed in HNSCC, wherein it serves as a tumor suppressor and inhibits tumor growth and metastasis [25]. *miR-196b-5p* expression upregulation in HNSCC has also been verified using TCGA database [26]. Although these authors described three different miRNAs, their study had fewer samples for verification or used different calculation methods to select miRNAs. Therefore, it was imperative to further verify the functions of these miRNAs. The miRNAs *miR-498-5p*, *miR-1-5p*, *miR-548f-3p*, and *miR-4714-3p* have not been reported in previous HNSCC-related studies; thus, additional research is warranted to determine their functions in HNSCC.

GO analysis revealed 11 DEMs that were primarily enriched at the nucleus, cytoplasm, and membrane and were related to the positive regulation of transcription from the RNA polymerase II promoter. These locations and pathways were associated with different physiological and pathological activities of the human body. Uncontrolled disease activity accelerates the occurrence and development of HNSCC, highlighting the importance of pathological changes related to these locations and pathways. Furthermore, the KEGG enrichment analysis revealed that the neurotrophin signaling pathway, protein processing in the endoplasmic reticulum, and microRNAs in cancer significantly affected HNSCC occurrence and development. We found that several targeted genes exert their functions through the neurotrophin signaling pathway, which performs key functions in the occurrence and development of HNSCC by regulating cell survival, angiogenesis, tissue invasion, DNA damage resistance, and epithelial-to-mesenchymal transition [27–30]. Gkouveris et al. demonstrated that interferon-gamma inhibits OSCC cell viability and migration and increases cellular apoptosis through the endoplasmic reticulum stress pathway [31]. Further, dentin sialophosphoprotein induces OSCC cell viability [32] and HIV protease inhibitors with radiosensitizing activities mediate HNSCC cell apoptosis [33] through the activation of the endoplasmic reticulum stress mechanisms. Overall, these pathways predicted herein may play an important role in HNSCC development and progression.

In the present study, we analyzed the miRNA expression levels and clinical information of 71 HNSCC patients and 13 normal subjects. A mess of samples would effectively reduce bias. The univariate CPHR and LASSO regression analyses yielded 11 DEMs. Thus, we constructed and systematically validated the risk signature and nomogram, which showed high predictive accuracy. In comparison with invasive tests or imageological examination, the risk signature and predicted nomogram were convenient and economic but must be verified in clinical studies and cell or animal experiments.

## Conclusion

We constructed an 11-miRNA risk signature and a nomogram that showed high correlation with HNSCC prognosis. The 3- and 5-year OS of patients with HNSCC could be accurately predicted through the risk signature and nomogram, thus serving as an effective tool for clinicians to facilitate decision-making.

## Methods

### Data resources and preprocessing

The miRNA sequencing data and related clinical information of patients with HNSCC were obtained from TCGA database (https://portal.gdc.cancer.gov/). The “edgeR” package in R software was used to identify DEMs from 797 miRNAs in 71 HNSCC samples and 13 normal samples based on |log2FC| ≥ 2 and adjusted *P*-value < 0.05. We then integrated the miRNA expression data and clinical information based on sample ID, and excluded four patients because they had no significant clinical date or their OS was less than 30 days to avoid introduction of mixed factors. Finally, 67 patients with HNSCC were enrolled as the primary cohort. In order to unbiasedly appraise the final model, 44 patients randomly selected from the primary cohort were designed as the internal validation cohort by using the “caret” package in R software.

### Construction of risk score formula

DEMs were detected from the univariate CPHR analysis of the primary cohort. These DEMs with a *P*-value < 0.05 were subjected to LASSO CPHR analysis to produce a miRNA signature using the “glmnet” package in R software. The risk score of each patient relied on the miRNA expression level and the regression coefficient obtained from the LASSO regression analysis. The risk score was calculated as follows: Risk score (miRNA-based classifier) = sum of coefficients × expression level of miRNAs, refer to previous description [34]. According to the median risk score value, 67 patients with HNSCC were divided into high-risk and low-risk groups.

### Assessment of miRNA signature

The survival rate between the high- and low-risk groups were compared via the Kaplan–Meier survival analysis. A time-dependent ROC curve analysis performed using “survival ROC” packages estimated the sensitivity and specificity of each miRNA signature on the basis of the AUC value.

The differences in the signature between predicted survival and actual survival were assessed and calibration curves were constructed. The C-index values were calculated to measure the predictive performance of the risk signature. All analyses were performed to compare the consistency between the primary and internal validated cohorts, refer to previous description [34].

### Construction and evaluation of a novel nomogram

Due to the T stage, M stage, N stage exist serious information loss, those clinical informations were excluded for guaranteeing the reality of this study. Risk signature and other clinical factors, such as age, sex, neoplasm histologic grade (grade), TNM stage, and Ragnum hypoxia score (hypoxia score), were included in the univariate and multivariate CPHR analyses of the primary cohort. Factors with a *P*-value < 0.05 both in the univariate and multivariate CPHR analyses were confirmed as independent prognostic variables and then used to establish a novel nomogram by using the package “rms” in R software. To evaluate the predictive ability of the novel nomogram, a calibration plot was constructed and AUC in ROC analysis and C-index were calculated. All analyses were implemented for the primary and internal validation cohorts.

### Functional enrichment analysis

Three online databases, including TargetScan, miRTarBase, and miRDB, were used to predict the potential target genes of prognostic miRNAs. The overlapping miRNA target genes predicted by these three databases were undergo to GO analysis and KEGG pathway enrichment analysis using DAVID (http://david.ncifcrf.gov/) refer to previous description [34]. *P* < 0.05 was considered the cut-off value.

## Data Availability

The datasets used and/or analyzed during the current study are available from the corresponding author on reasonable request.

## Abbreviations

HNSCC: Head and neck squamous cell carcinoma
miRNA: microRNA
GO: Gene Ontology
KEGG: Kyoto Encyclopedia of Genes and Genomes
DEM: Differentially expressed miRNA
OS: Overall survival
TCGA: The Cancer Genome Atlas
CPHR: Cox proportional hazard regression
OSCC: Oral squamous cell carcinoma
